# A Novel Classification System to Address Financial Impact and Referral Decisions for Bile Duct Injury in Laparoscopic Cholecystectomy

**DOI:** 10.1155/2011/371245

**Published:** 2011-09-08

**Authors:** Robert M. Cannon, Guy Brock, Joseph F. Buell

**Affiliations:** ^1^Department of Surgery, University of Louisville, Louisville, KY 40202, USA; ^2^Department of Bioinformatics and Biostatistics, School of Public Health & Information Sciences, University of Louisville, Louisville, KY 40202, USA; ^3^Tulane Abdominal Transplant Institute, Department of Surgery, Tulane University, New Orleans, LA 70112, USA

## Abstract

*Purpose*. The study was undertaken to evaluate a novel classification system developed to estimate financial cost of bile duct injury (BDI) and to aid in decision making for referral. *Study Design*. A retrospective review of patients referred for BDI was performed. Grade I injuries involve the duct of Luschka or accessory right hepatic ducts, grade II includes all other biliary injuries, and grade III includes all vasculobiliary injuries. Groups were compared using standard statistical methods. *Results*. There were 14 grade I, 74 grade II, and 20 grade III injuries. There was a significant difference in the cost and mortality of grade I ($12,457, 0%), grade II ($46,481, 1.4%), and grade III ($69,368, 15%, *P* = 0.002 and *P* = 0.030, resp.) injuries. Grade II and III injuries were significantly more likely to require surgical repair (OR 27.7, *P* < 0.001). *Conclusion*. We have presented a simple classification system that is able to accurately predict cost and need for surgical repair.

## 1. Introduction

Laparoscopic cholecystectomy is one of the most common general surgical procedures performed in the United States today. Whether performed as an open or laparoscopic procedure, bile duct injury (BDI) is a well-recognized and feared complication [1–5]. Bile duct injuries often occur in the setting of distorted anatomy, especially in the presence of acute cholecystitis or excessive bleeding [1, 5–7]. With the introduction of laparoscopic cholecystectomy, the incidence of bile duct injury has been reported to be roughly 0.5% [8–10]. This is nearly double the reported incidence of 0.1–0.25% seen with open cholecystectomy [11–13]. Despite attempts to decrease this rate through operative maneuvers and improved teaching processes, this incidence has not decreased markedly [14–16]. 

Few bile duct injuries are identified in the operating room. The majority of patients present with jaundice over the subsequent week following their cholecystectomy. The diagnostic procedure of choice has become endoscopic retrograde cholangiopancreatography (ERCP). Several groups have devised classification systems that attempt to convey mechanism and level of injury, as well as the complexity of repair that will be required. The system proposed by Bismuth and Majno was based on the level of chronic biliary strictures [17]. The Stewart and Way system addressed acute injuries and incorporated mechanism of injury [18]. Strasberg et al. unified these two systems and classified injury based on anatomic location and the complexity of repair required [19]. This system is relatively complex but defines injuries in a very precise manner. 

A relatively recent addition to classification systems has been consideration of concomitant arterial injuries. Of the over 600 publications on bile duct injury, relatively few have discussed the significance of concomitant arterial injuries [20–26]. The Hannover group has incorporated the presence of arterial injury, resulting in a more accurate and considerably more complex system [27]. The drawback to the precision of the currently proposed systems is their high degree of complexity, which makes their routine incorporation into clinical use difficult.

One area that has yet to be addressed by current classification schemes is the financial impact of bile duct injury following laparoscopic cholecystectomy. We have devised a simple, three-tier classification scheme with the primary goal of stratifying injuries based on the financial cost of definitive management. Secondary endpoints assessed in this study include need for operative repair, risk of mortality, and biliary stenosis following definitive management.

## 2. Patients and Methods

Between 1992 and 2010, a total of 108 bile duct injuries were managed by our hepatobiliary group. With institutional review board approval, a retrospective review of these cases was undertaken. Patient demographics, operative procedures, complications, mortality, and financial costs of care were examined. A three-tier classification system was devised to stratify injuries. Grade I injuries consisted of leaks from the cystic duct stump, duct of Luschka, or accessory right hepatic ducts. Grade II injuries consisted of all other levels of biliary injury, including those to the common bile duct or intrahepatic bile ducts. Grade III includes all combined vascular and biliary injuries. The primary endpoint of this study was to assess the ability of this classification system to stratify patients based on total financial cost of definitive management. Other endpoints assessed included need for surgical repair, mortality, and biliary restenosis.

Management of minor biliary injuries such as leaking Duct of Luschka consisted primarily of ERCP with temporary stenting of the common bile duct. For more complex injury patterns, ERCP played a primarily diagnostic role. Any fluid collections were percutaneously drained to control biliary sepsis, and patients were started on broad spectrum antibiotic therapy. Arterial imaging was employed with visceral angiography or more recently CT angiography. Injuries at Bismuth level II or higher were initially managed by placement of PTC catheters. Extensive injuries often necessitated the placement of multiple catheters. Definitive surgical intervention was then planned based on radiographic level of injury. Patients with complex injuries were typically treated with two weeks of conservative management prior to definitive surgical therapy. 

The primary surgical therapy employed consisted of debridement of the damaged bile duct followed by retrocolic Roux-en-Y hepaticojejunostomy to the remaining biliary radicles. These were drained with the use of PTC catheters. Small undrained biliary radicles greater than 5 mm were stented with a segment of pediatric feeding tube. For injuries extending high into segment IV, a Kasai portoenterostomy was performed. In cases of extensive unilateral biliary and/or vascular injury, major hepatic resection was performed. 

Statistical analysis was performed to compare patient demographics, complications following definitive management, mortality, late biliary stricture, length of stay, and financial cost between the three grades of injury. Financial costs included hospitalization, OR and surgeon's fees, and outpatient pharmacy charges. Continuous variables were analyzed using the Wilcoxon-Mann-Whitney or Kruskal-Wallis test where appropriate. Late restricture, complications, and mortality were analyzed using Fisher's exact test and logistic regression. The necessity for surgical versus endoscopic or percutaneous management based on grade of injury, as well as the impact of repair attempts at the referring institution on long-term biliary stricture, was analyzed using logistic regression and Fisher's exact test. A significance level of *P* < 0.05 was set.

## 3. Results

There were 108 patients referred for evaluation of bile duct injuries during the study period. The group consisted of 79 women and 29 men, at an average age of 46.9 years. Injuries were recognized intraoperatively in 32 patients. Additional diagnostic measures included ERCP (58.3%) and HIDA (3.7%). The median interval from laparoscopic cholecystectomy to recognition of the injury was 3 days. There were 20 combined vasculobiliary injuries (Table 1). 

Attempts at therapy were made at the primary institution in 28 patients. These included attempted hepaticojejunostomy (*n* = 15), choledochojejunostomy (*n* = 6), primary repair (*n* = 3), and T-tube placement (*n* = 4). Attempted surgical therapy at the primary institution was associated with a significantly higher incidence of restenosis following definitive management (OR: 7.0, 95% CI: 2.5–19.6, *P* < 0.001). 

Definitive therapy at our institution consisted of biliary enteric anastomosis, portoenterostomy, ERCP with stenting, hepatic resection, and percutaneous procedures (Table 2). Twenty-one patients suffered postrepair biliary stricture at a median of 6 months. There were four deaths in this series. Three were from sepsis with multisystem organ failure, and the other was from postoperative pulmonary embolus. The median followup after definitive management was 90 months. 

As described above, a three-tier classification system was devised in an attempt to stratify the overall financial impact of bile duct injury. There were 14 patients with grade I injuries, which were either repaired at the primary operation or managed with endoscopic stenting (Table 3). There were no deaths or late biliary strictures in this group, while one patient suffered a complication. The mean length of stay following referral was 1.6 days, with an average cost of $12,457.

Grade II injuries were present in 74 patients. Operative repair was required in 66 patients, while the rest were managed with endoscopic or percutaneous measures. Complications occurred in 8 patients, and there was 1 death from sepsis. Late biliary strictures occurred in 17 patients. Average length of stay was 8.8 days, with a mean total cost of $46,481. Application of the Hannover [27] system to patients with grade II injuries provided no ability to discriminate based on the probability of late restenosis after definitive management (*P* = 0.639), total cost (*P* = 0.423), or length of stay (*P* = 0.054). There were significant differences in the method of definitive management based on the Hannover classification in this group (*P* = 0.003). In particular, patients with class B or C lesions (tangential injury or occlusion of the bile duct with a clip) were more likely to have nonoperative management with ERCP or PTC (37.5% versus 2.4%; *P* < 0.001). In contrast, patients with lesions above the bifurcation of the hepatic duct (C4, D4, or E4) were significantly more likely to require hepatic resection for definitive management (20.0% versus 1.9%; *P* = 0.039). The odds of requiring hepatic resection for patients with lesions above versus below the hepatic bifurcation were 12.235 (95% CI: 1.278–117.094, *P* = 0.030).

Combined vasculobiliary injuries (grade III) occurred in 20 patients. More proximal biliary injuries as defined by the Bismuth classification were significantly more likely to be associated with a concomitant vascular injury (OR: 1.8, 95% CI: 1.1–2.8, *P* = 0.011). All but 2 patients required operative repair, although no vascular reconstructions were necessary. There were six complications in this group, including sepsis (2), biliary leak (2), pulmonary embolism (2), and dehydration (2). There were three deaths. Two were from sepsis with multiple organ failure while the last was from pulmonary embolism in the outpatient setting. There were 4 late strictures in this group. Length of stay was an average of 11.8 days, with a mean total cost of $69,368.

Application of the Hannover system to patients with grade III injuries provided significant ability to discriminate based on the percentage of patients who had restenosis of the bile duct following definitive management (*P* = 0.024). Specifically, patients with injuries to the proper hepatic artery (Hannover Cp, Dp, Ep) had a 100% incidence of late restenosis compared to 5.9% of patients with injuries to other vessels (*P* = 0.004). The Hannover system was also able to provide information as the required modality of repair for patients with grade III injuries (*P* = 0.013). Specifically, 66.7% of patients with injuries to the portal vein were able to undergo nonoperative management, compared to none of the patients with arterial injuries (*P* = 0.018).

Analysis of the primary endpoint of total cost revealed significant differences between the three groups in aggregate (*P* < 0.001). Between-group comparisons revealed significantly higher cost for grade II versus grade I injuries (*P* = 0.002) and for grade III versus grade II injuries (*P* = 0.002). There were no significant differences between the three groups in age, gender, or time to injury recognition (Table 4).

 The classification system was not able to discriminate based on the incidence of restenosis (*P* = 0.109) or complications following definitive management (*P* = 0.107). Length of stay was predictably longer with increasing grade of injury (*P* < 0.001). As grade of injury increased, there were a significantly higher odds that surgical (as opposed to endoscopic or percutaneous) therapy would be required for management (*P* < 0.001). For grade II versus I injuries, odds of requiring surgical therapy were 27.7 (95% CI: 5.5–138.9, *P* < 0.001). There was no significant difference in the requirement for operative therapy between grade III and grade II injuries (OR: 1.8, 95% CI: 0.4–9.0, *P* = 0.450). There were significant differences in mortality between the three grades of injury in aggregate (*P* = 0.030) (Table 4).

## 4. Discussion

Bile duct injuries with laparoscopic cholecystectomy continue to occur at nearly twice the rate encountered with open cholecystectomy. The United States has recently witnessed multiple attempts by the federal government at medical cost containment, including proposed withholding of payment for the management of iatrogenic complications. To date, multiple systems for classification of bile duct injury have been devised to define anatomy, mechanism of injury, and expected complexity of repair; however, none address the financial cost of management [17–19, 27]. 

These systems have evolved from Bismuth's description of chronic bile duct strictures to the current complex, multi-level systems incorporating mechanism of injury, arterial injury, and expected mode of repair. The importance of vasculobiliary injury has been highlighted by reports of increased mortality, liver ischemia, and repair failures when vascular injury is added to biliary injury [21, 24, 25]. Several authors have reported the incidence of vascular injury with biliary injury to be up to 20% [28–30]. The presence of vasculobiliary injury significantly increases the complexity of repair, including procedures such as hepatic resection, Kasai portoenterostomy, or even liver transplantation [31–34].

Our current series has identified a nearly 20% incidence of vascular injury in the setting of biliary injury following laparoscopic cholecystectomy. This is consistent with the published literature; however, we recognize that this incidence may be overestimated in that we only saw cases deemed severe enough for referral to a tertiary center. We identified proximal biliary injury as a significant risk factor for concomitant vascular injury. Although the presence of any vascular injury in our series was not significantly associated with risk of late stricture after repair, we did show an increased risk of mortality. Injuries to the main hepatic artery specifically, however, are universally associated with late restenosis after repair. As the sole factor defining a grade III injury, vascular injury also significantly increases the cost of definitive management. 

Although we developed this grading system to assess the financial impact of bile duct injury, perhaps a more important attribute of our scheme is its simplicity of use in helping practicing surgeons make decisions on referral. The currently available systems of bile duct injury classification do an excellent job of precisely defining anatomy and mechanism of injury; however, they are too complex and cumbersome to be remembered offhand and routinely applied in routine practice. Two findings in this study are worth emphasizing. First is, that, when attempts at surgical repair are made in the referring institution, the odds of late biliary stricture following definitive management significantly increase. The other is that patients with grade II or III injuries are significantly more likely to require operative therapy to repair their biliary tree. This is particularly true when the vascular injury is to one of the hepatic arteries rather than to the portal vein. 

**Figure 1:**
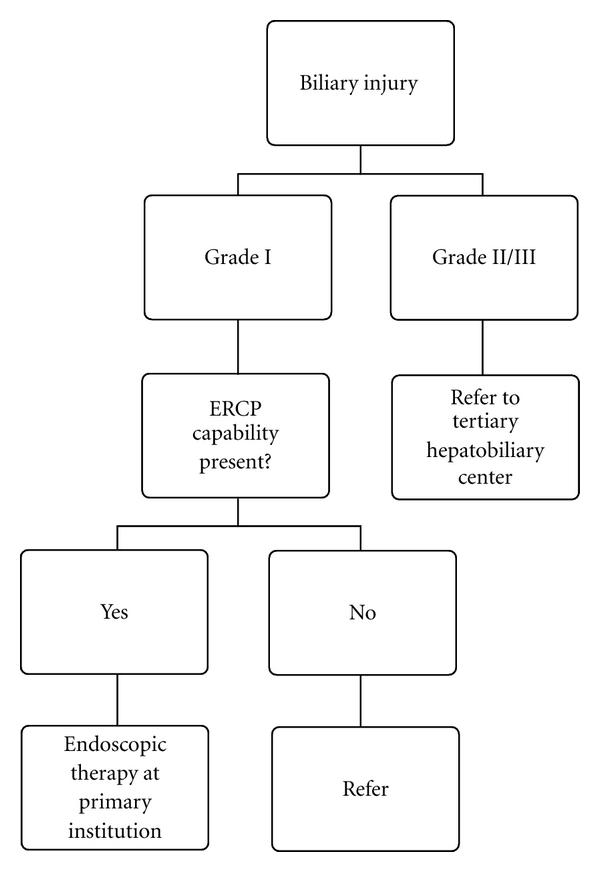
Proposed decision tree for referral of bile duct injuries based on the proposed three-tier system.

Based on these findings, we propose the following decision tree when biliary injury is diagnosed following laparoscopic cholecystectomy (Figure 1). For grade I injuries, endoscopic treatment is likely all that will be required, and these can be managed safely at the primary center if ERCP capability is present. Patients with grade II or III injuries represent a significantly more complex problem and should be referred immediately to a tertiary hepatobiliary center. Attempts at surgical repair by surgeons inexperienced with complex biliary surgery should be avoided in these cases given the increased risk for later complications. For patients with grade II injuries not involving stricture or complete common bile duct transection (Hannover class B and C), nonoperative management remains a possibility. More extensive grade II injuries (Hannover class D and E) almost universally require operative management. For patients with grade II injuries above the hepatic bifurcation, there is a significantly greater likelihood of requiring hepatic resection for definitive therapy. For patients with grade III injuries involving the portal vein, there still remains the potential for nonoperative management, either through ERCP with stenting or PTC. All other patients with grade III injuries should undergo operative biliary reconstruction and/or hepatic resection. Grade III injuries to the main hepatic artery were all associated with late restenosis, a factor which should be discussed thoroughly with the patient preoperatively.

Here we have presented a novel classification system for biliary injury that is the first to address the financial costs of definitive management. While this system does not provide the precise anatomic and mechanistic information afforded by current classification schemes, we believe its simplicity makes it applicable to routine clinical practice. This is especially important as it applies to the decision making tree in referring patients with BDI to experienced hepatobiliary centers. Application of a minimal amount of additional anatomic information to this scheme (particularly whether vascular injury is to the portal vein or main hepatic artery, whether the biliary injury is above or below the bifurcation, and whether the injury involves complete transection or stenosis of the common bile duct) adds significant information as to the type of management strategy required. This study is limited by its retrospective design and confinement to a tertiary referral center. Prospective validation of this system's utility in a broader range of clinical settings will be required.

##  Disclosure

The authors have no disclosures to make.

## Figures and Tables

**Table 1:** Overall cohort characteristics.

Gender	
Male	29 (26.9%)
Female	79 (73.8%)
Age (mean, range)	46.9 (15–85)
Acute cholecystitis	34 (31.5%)
Conversion	34 (31.5%)
Intraoperative recognition of injury	32 (30.2%)
Vasculobiliary injury	20 (18.5%)
Vessel involved	
Right hepatic	12 (60.0%)
Left hepatic	1 (5.0%)
Main hepatic	3 (15.0%)
Celiac	1 (5.0%)
Portal vein	3 (15.0%)
Time to diagnosis (days, median, range)	3 (0–1000)
Complications after definitive therapy	15 (13.9%)
Deaths	4 (3.7%)
Restenosis after repair	21 (19.4%)
Total cost (mean, range)	$46,519 ($1,795–$171,814)

**Table 2:** Definitive therapy after referral.

Mode of definitive management	
ERCP with Stenting	19 (17.6%)
Percutaneous	7 (6.5%)
Choledochojejunostomy	1 (0.9%)
Hepaticojejunostomy	68 (63.0%)
Hepatic Resection	7 (6.5%)
Kasai Portoenterostomy	3 (2.8%)
No further therapy	3 (2.8%)

**Table 3:** Therapeutic measures by grade of injury. CJ: choledochojejunostomy; HJ: hepaticojejunostomy.

Grade	Endoscopic	Percutaneous	CJ	HJ	Resection	Kasai	None
I	11 (78.6%)	1 (7.1%)	0	0	0	0	2 (14.3%)
II	7 (9.5%)	5 (6.8%)	1 (1.4%)	54 (73.0%)	5 (6.8%)	1 (1.4%)	1 (1.4%)
III	1 (5.0%)	1 (5.0%)	0	14 (70.0%)	2 (10.0%)	2 (10.0%)	0

**Table 4:** Comparison between grades of injury.

	Grade I	Grade II	Grade III	*P* value
Gender				
Male	5 (35.7%)	17 (23.0%)	7 (35.0%)	
Female	9 (64.3%)	57 (77.0%)	13 (65.0%)	0.328
Age (mean)	46.2	44.9	54.5	0.130
Days to recognition of injury (median)	2.5	3.0	2.5	0.796
Length of stay (days, mean)	1.6	8.8	11.8	**<0.001**
Complications	1 (7.1%)	8 (10.8%)	6 (30.0%)	0.107
Late restenosis	0	17 (23.0%)	4 (20.0%)	0.109
Mortality	0	1 (1.4%)	3 (15.0%)	**0.030**
Cost (mean)	$12,457	$46,481	$69,368	**<0.001**

## References

[1] Strasberg SM (2008). Error traps and vasculo-biliary injury in laparoscopic and open cholecystectomy. *Journal of Hepato-Biliary-Pancreatic Surgery*.

[2] Strasberg SM, Sanabria JR, Clavien PA (1992). Complications of laparoscopic cholecystectomy. *Canadian Journal of Surgery*.

[3] Perissat J, Collet D, Edye M, Magne E, Belliard R, Desplantez J (1992). Laparoscopic cholecystectomy: an analysis of 777 cases. *Bailliere’s Clinical Gastroenterology*.

[4] Fletcher DR, Hobbs MST, Tan P (1999). Complications of cholecystectomy: risks of the laparoscopic approach and protective effects of operative cholangiography: a population-based study. *Annals of Surgery*.

[5] Regöly-Mérei J, Ihász M, Szeberin Z, Sándor J, Máté M (1998). Biliary tract complications in laparoscopic cholecystectomy: a multicenter study of 148 biliary tract injuries in 26,440 operations. *Surgical Endoscopy*.

[6] Strasberg SM (1997). Cholelithiasis and acute cholecystitis. *Bailliere’s Clinical Gastroenterology*.

[7] Strasberg SM (1999). Laparoscopic biliary surgery. *Gastroenterology Clinics of North America*.

[8] MacFadyen BV, Vecchio R, Ricardo AE, Mathis CR (1998). Bile duct injury after laparoscopic cholecystectomy: the United States experience. *Surgical Endoscopy*.

[9] Targarona EM, Marco C, Balagué C (1998). How, when, and why bile duct injury occurs: a comparison between open and laparoscopic cholecystectomy. *Surgical Endoscopy*.

[10] Archer SB, Brown DW, Smith CD, Branum GD, Hunter JG (2001). Bile duct injury during laparoscopic cholecystectomy: results of a national survey. *Annals of Surgery*.

[11] Andren-Sandberg A, Alinder G, Bengmark S (1985). Accidental lesions of the common bile duct at cholecystectomy. Pre- and perioperative factors of importance. *Annals of Surgery*.

[12] Gilliland TM, Traverso LW (1990). Modern standards for comparison of cholecystectomy with alternative treatments for symptomatic cholelithiasis with emphasis on long term relief of symptoms. *Surgery Gynecology and Obstetrics*.

[13] McSherry CK (1989). Cholecystectomy: the gold standard. *American Journal of Surgery*.

[14] Katkhouda N, Mavor E, Mason RJ (2000). Visual identification of the cystic duct-CBD junction during laparoscopic cholecystectomy (visual cholangiography): an additional step for prevention of CBD injuries. *Surgical Endoscopy*.

[15] Ahrendt SA, Pitt HA (2001). Surgical therapy of iatrogenic lesions of biliary tract. *World Journal of Surgery*.

[16] Tomonaga T, Filipi CJ, Lowham A, Martinez T (1999). Laparoscopic intracorporeal ultrasound cystic duct length measurement: a new technique to prevent common bile duct injuries. *Surgical Endoscopy*.

[17] Bismuth H, Majno PE (2001). Biliary strictures: classification based on the principles of surgical treatment. *World Journal of Surgery*.

[18] Way LW, Stewart L, Gantert W (2003). Causes and prevention of laparoscopic bile duct injuries: analysis of 252 cases from a human factors and cognitive psychology perspective. *Annals of Surgery*.

[19] Strasberg SM, Callery MP, Soper NJ (1996). Laparoscopic surgery of the bile ducts. *Gastrointestinal Endoscopy Clinics of North America*.

[20] Majno PE, Prêtre R, Mentha G, Morel P (1996). Operative injury to the hepatic artery: consequences of a biliary-enteric anastomosis and principles for rational management. *Archives of Surgery*.

[21] Gupta N, Solomon H, Fairchild R, Kaminski DL (1998). Management and outcome of patients with combined bile duct and hepatic artery injuries. *Archives of Surgery*.

[22] Bachellier P, Nakano H, Weber JC (2001). Surgical repair after bile duct and vascular injuries during laparoscopic cholecystectomy: when and how?. *World Journal of Surgery*.

[23] Mathisen Ø, Søreide O, Bergan A (2002). Laparoscopic cholecystectomy: bile duct and vascular injuries: management and outcome. *Scandinavian Journal of Gastroenterology*.

[24] Koffron A, Ferrario M, Parsons W, Nemcek A, Saker M, Abecassis M (2001). Failed primary management of iatrogenic biliary injury: incidence and significance of concomitant hepatic arterial disruption. *Surgery*.

[25] Buell JF, Cronin DC, Funaki B (2002). Devastating and fatal complications associated with combined vascular and bile duct injuries during cholecystectomy. *Archives of Surgery*.

[26] Tzovaras G, Dervenis C (2007). Vascular injuries in laparoscopic cholecystectomy: an underestimated problem. *Digestive Surgery*.

[27] Bektas H, Schrem H, Winny M, Klempnauer J (2007). Surgical treatment and outcome of iatrogenic bile duct lesions after cholecystectomy and the impact of different clinical classification systems. *British Journal of Surgery*.

[28] Stewart L, Way LW, Meyers WC (1995). Bile duct injuries during laparoscopic cholecystectomy: factors that influence the results of treatment. *Archives of Surgery*.

[29] Frilling A, Li J, Weber F (2004). Major bile duct injuries after laparoscopic cholecystectomy: a tertiary center experience. *Journal of Gastrointestinal Surgery*.

[30] Schmidt SC, Langrehr JM, Hintze RE, Neuhaus P (2005). Long-term results and risk factors influencing outcome of major bile duct injuries following cholecystectomy. *British Journal of Surgery*.

[31] Pickleman J, Marsan R, Borge M (2000). Portoenterostomy: an old treatment for a new disease. *Archives of Surgery*.

[32] Laurent A, Sauvanet A, Farges O, Watrin T, Rivkine E, Belghiti J (2008). Major hepatectomy for the treatment of complex bile duct injury. *Annals of Surgery*.

[33] Robertson AJ, Rela M, Karani J, Steger AC, Benjamin IS, Heaton ND (1998). Laparoscopic cholecystectomy injury: an unusual indication for liver transplantation. *Transplant International*.

[34] Nishio H, Kamiya J, Nagino M (1999). Right hepatic lobectomy for bile duct injury associated with major vascular occlusion after laparoscopic cholecystectomy. *Journal of Hepato-Biliary-Pancreatic Surgery*.

